# ACE2 maybe serve as a prognostic biomarker in breast invasive carcinoma

**DOI:** 10.1002/jcla.24362

**Published:** 2022-04-04

**Authors:** Jie ling, Ning Peng, Lifei luo

**Affiliations:** ^1^ Department of Clinical Laboratory Taizhou First People’s Hospital Huangyan Hospital of Wenzhou Medical University Taizhou China; ^2^ Department of Clinical Laboratory Taizhou Hospital of Zhengjiang Province affiliated of Wenzhou Medical University Linhai China; ^3^ Department of Clinical Laboratory Taizhou Enze Medical Center (Group) Enze Hospital Taizhou China

**Keywords:** ACE2, breast cancer, immune infiltration, prognostic biomarker

## Abstract

**Background:**

Breast cancer is a frequently occurring malignant tumor in women. Angiotensin‐converting enzyme 2 (ACE2) is widely expressed in most organs; however, the association of ACE2 with prognosis and immune infiltration in breast invasive carcinoma (BRCA) remains elusive.

**Methods:**

We explored the expression level and prognostic value of ACE2 in patients with BRCA using a series of online bioinformatics analysis databases encompassing Oncomine, UALCAN, Kaplan–Meier plotter, TIMER, LinkedOmics, and GEO. qRT‐PCR was performed to verify our findings.

**Results:**

Angiotensin‐converting enzyme 2 mRNA and protein expression levels were decreased in BRCA tissues, and patients with low ACE2 expression levels had a poor prognosis. DNA promoter methylation of *ACE2* significantly downregulated ACE2 expression in BRCA, while the expression of this protein was positively linked to immune infiltration of B cells, CD8^+^ and CD4^+^ T cells, neutrophils, and dendritic cells in BRCA tissues. The high expression level of ACE2 in enriched basophils, CD8^+^ T cells, and type‐2 helper T cells, which showed decreasing levels, indicated a better prognosis for BRCA. Enrichment analyses revealed that NF‐κB, IL‐17, and TNF signaling pathways were highly correlated to ACE2 in BRCA. Verification study revealed that downregulation of ACE2 was associated with a better prognosis in BRCA. Univariate and multivariate analysis confirmed ACE2 expression and clinical stage as independent prognostic factors for breast cancer.

**Conclusions:**

Angiotensin‐converting enzyme 2 may be a potential prognostic biomarker and target for BRCA. Nevertheless, future investigations are needed for validating our findings and promoting the clinical application of ACE2 in BRCA.

## Graphical abstract

Breast cancer is a frequently occurring malignant tumor in females. Angiotensin‐converting enzyme 2 (ACE2) is widely expressed in most organs; however, the association of ACE2 with prognosis and immune infiltration in breast invasive carcinoma (BRCA) remains elusive. We explored the expression level and prognostic value of ACE2 in patients with BRCA using a series of online bioinformatics analysis databases encompassing Oncomine, UALCAN, Kaplan–Meier plotter, TIMER, LinkedOmics, and GEO. qRT‐PCR was performed to verify our findings. ACE2 mRNA and protein expression levels were decreased in BRCA tissues, and patients with low ACE2 expression levels had a poor prognosis. DNA promoter methylation of *ACE2* significantly downregulated ACE2 expression in BRCA, while the expression of this protein was positively linked to immune infiltration of B cells, CD8^+^ and CD4^+^ T cells, neutrophils, and dendritic cells in BRCA tissues. The high expression level of ACE2 in enriched basophils, CD8^+^ T cells, and type‐2 helper T cells, which showed decreasing levels, indicated a better prognosis for BRCA. Enrichment analyses revealed that NF‐κB, IL‐17, and TNF signaling pathways were highly correlated to ACE2 in BRCA. Verification study revealed that downregulation of ACE2 was associated with a better prognosis in BRCA. Univariate and multivariate analysis confirmed ACE2 expression and clinical stage as independent prognostic factors for breast cancer. ACE2 may be a potential prognostic biomarker and target for BRCA. Nevertheless, future investigations are needed for validating our findings and promoting the clinical application of ACE2 in BRCA.

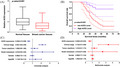

## 1 INTRODUCTION

Breast cancer mostly occurs in women and is associated with high mortality. Classically, the prognosis of breast cancer correlates with age, stage, tumor type, tumor grade, and lymphovascular status.^1^ According to Global Cancer Statistics 2018, breast cancer accounted for 11.6% of cases, being the second‐largest commonly diagnosed cancer.^2^ *BRCA1* and *BRCA2* genes are tumor suppressor genes that help repair DNA damage to suppress breast cancer tumorigenesis. People with mutations in *BRCA* genes are vulnerable to breast cancer. Statistics show that *BRCA1* and *BRCA2* mutations could increase the susceptibility of 80‐year‐olds individuals by 72% and 69%, respectively.^3^ The treatment options available for noninvasive and invasive breast cancer are complicated and diverse. The NCCN guidelines for breast cancer treatment encourage HR‐positive, HER2‐negative early invasive breast cancer patients to undergo testing using multigene assays with or without adjuvant systemic therapy based on nodal status and other clinicopathological features.^4^ Invasive ductal breast cancer (IDC) and invasive lobular breast cancer (ILC) are the most frequent histological subtypes of invasive breast cancer. ER‐positive, HER2‐negative patients with IDC or ILC show differences in immune infiltration in the light of clinical relevance, prevalence location, among other factors.^5^

Angiotensin‐converting enzyme 2 balances the function of ACE. Most organs, including lungs, intestines, heart, arteries, and kidneys, as well as the female reproductive system, express ACE2.^6^ Furthermore, in the cardiovascular system, ACE2 can protect the heart and relax blood vessels. ACE2 also plays important roles in the renal and reproductive system.^7^ ACE2 deficiency results in hypertension, diabetes, and several cardiovascular diseases.^8^ Recently, ACE2 has been reported to protect breast cancer patients. One possible mechanism of ACE2 to suppress the carcinogenesis of breast cancer can be the inhibition of the VEGFa/VEGFR2/ERK pathway.^9^ However, the complete mechanism of how ACE2 acts in breast cancer, especially BRCA, remains unclear.

In this work, we evaluated ACE2 mRNA and protein expression, as well as promoter methylation levels using the Oncomine and UALCAN databases. BRCA survival analysis was performed in the Kaplan–Meier plotter database (KMPD). The relationship between ACE2 expression and immune infiltration and immune cell gene biomarkers was explored in Tumor Immune Assessment Resource (TIMER) database. Furthermore, Gene Set Enriching Analysis (GSEA) was utilized to perform the GO and KEGG enrichment analyses using the LinkedOmics database.

## 2 MATERIALS AND METHODS

### 2.1 Oncomine

Oncomine contains 715 datasets and 86,733 samples, being the most comprehensive online cancer gene chips database worldwide.^10^ The expression of ACE2 in BRCA and other tumors was studied in Oncomine (https://www.oncomine.org/resource/main.html). The threshold was a *p*‐value of 1E‐4, a twofold change, and a top 10% of the gene ranking.

### 2.2 UALCAN

UALCAN is a comprehensive database providing diverse in‐depth analysis of TCGA expression data (http://ualcan.path.uab.edu/cgi‐bin/ualcan‐res.pl).^11^ The TCGA BRCA dataset (*N* = 537) in the “Expression Analysis” module in TCGA was adopted for assessing *ACE2* mRNA levels in various subgroups of BRCA patients.

### 2.3 Kaplan–Meier plotter (KM‐plotter)

Kaplan–Meier plotters analyze the influence of various genes on the survival of patients with certain types of cancers, including breast, ovarian, lung, and gastric cancer (https://kmplot.com/analysis/).^12^ The TCGA BRCA dataset was obtained by selecting “breast cancer” from the Pan‐cancer RNA‐seq module. We analyzed the association of *ACE2* expression with overall survival (OS) and relapse‐free survival (RFS) in BRCA patients (*n =* 1090) using a KM survival plot. Furthermore, the correlation of ACE2 expression and the RFS of BRCA patients with different clinicopathological features was analyzed. Patients were split based on "automatically select the best cut‐off point," and the follow‐up threshold covered all patients.

### 2.4 Tumor Immune Assessment Resource (TIMER)

Tumor immune assessment resource tool is used for investigating correlations between immune infiltration and TCGA tumor (https://cistrome.shinyapps.io/timer/).^13^ We used TIMER database Gene and SCNA modules to evaluate the correlation between *ACE2* expression and immune infiltration and gene biomarkers of immune cells using the TCGA BRCA dataset (*N* = 537).

### 2.5 LinkedOmics

The LinkedOmics database is an online platform that helps analyze 32 types of cancer and 11,158 patients from TCGA (http://www.linkedomics.org/login.php) ^14^. Differentially expressed genes that correlated to ACE2 expression were determined by the LinkFinder module (*n =* 1093). The Spearman test was used to analyze the results statistically. In the LinkInterpreter module, GSEA (Gene Set Enriching Analysis) tool was utilized for carrying out GO (CC, BP, and MF), KEGG pathway, kinase‐target enrichment, miRNA‐target enrichment, and transcription factor enrichment analyses. The rank criteria were based on *p*‐value from LinkFinder results. The minimal gene size was 3 and 500 simulations were tested.

### 2.6 Clinical specimens

After obtaining informed consent from patients of Enze Hospital, Taizhou Enze Medical Center (Group), breast cancer tissues and pair‐normal breast tissues (*n =* 52) of these patients were used for the study. Our study was approved and supported by the ethics committee of Enze Hospital, Taizhou Enze Medical Center (Group). None of the patients received local or systemic treatment preoperatively.

### 2.7 qRT‐PCR

TRIzol reagent (Vazyme) was used to isolate total RNA. The synthesis of cDNAs corresponding to the mRNAs of interest was conducted using PrimeScript RT‐polymerase (Vazyme), SYBR‐Green Premix (Vazyme), and specific PCR primers (Sangon). Glyceraldehyde‐3‐phosphate dehydrogenase was used as an internal control. The 2^−ΔΔCt^ method was used to calculate fold‐changes. The difference in the expression of *ACE2* and the prognosis of ACE2 in breast cancer were explored with Student's *t* test and Kaplan–Meier analysis.

## 3 RESULTS

### 3.1 ACE2 expression in BRCA

**FIGURE 1:**
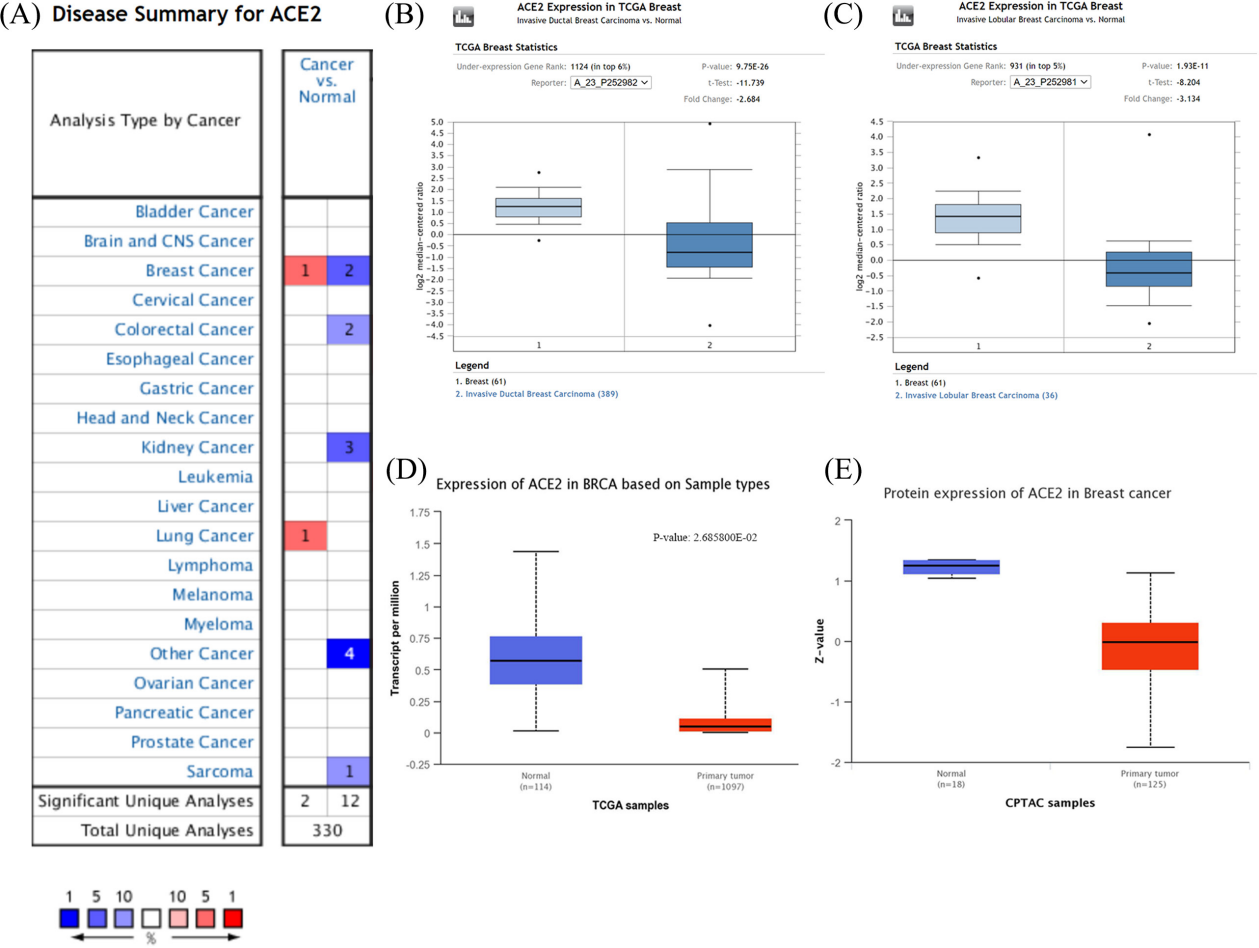
The mRNA and protein expression of ACE2 in breast invasive carcinoma (BRCA). (A) ACE2 expression variations in different cancers and normal tissues analyzed via the Oncomine database. (B) *ACE2* mRNA expression in Invasive Ductal Breast Cancer (Oncomine). (C) *ACE2* mRNA expression in Invasive Lobular Breast Cancer (Oncomine). (D) *ACE2* mRNA expression in BRCA analyzed via the UALCAN database. (E) ACE2 protein expression in BRCA (UALCAN)

**FIGURE 2:**
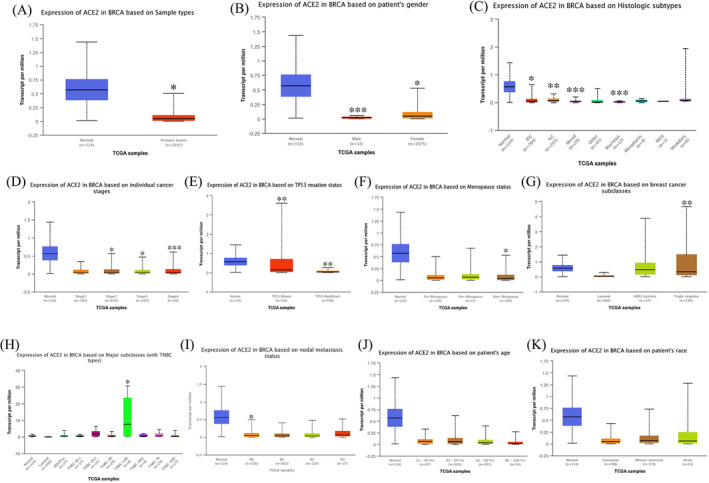
Angiotensin‐converting enzyme 2 mRNA expression based on various clinicopathological characteristics (UALCAN). (A) Relative expression of ACE2 in normal and BRCA samples. (B) Relative expression of ACE2 in normal individuals or male or female BRCA patients. (C) Relative expression of ACE2 in normal individuals or patients with IDC (Infiltrating Ductal Carcinoma), ILC (Infiltrating Lobular Carcinoma), Mixed histology, Mucinous Carcinoma, Medullary Carcinoma, Infiltrating Carcinoma NOS, Metaplastic Carcinoma, or other BRCA patients. (D) Relative expression of ACE2 in normal individuals or stages 1, 2, 3, or 4 BRCA patients. (E) Relative expression of ACE2 in normal individuals or TP53‐Mutant or TP53‐NonMutant BRCA patients. (F) Relative expression of ACE2 in normal individuals or Pre‐Menopause, Peri‐Menopause, or Post‐Menopause BRCA patients. (G) Relative expression of ACE2 in normal individuals or Luminal, HER2 positive, or Triple‐negative BRCA patients. (H) Relative expression of ACE2 in normal individuals or Luminal, HER2 positive, TNBC‐BL1 (TNBC Basal‐like 1), TNBC‐BL2 (TNBC Basal‐like 2), TNBC‐IM (TNBC Immunomodulatory), TNBC‐M (TNBC Mesenchymal), TNBC‐MSL (TNBC mesenchymal stem‐like), TNBC‐LAR (TNBC luminal androgen receptor), or TNBC‐UNS (TNBC unspecified) BRCA patients. (I) Relative expression of ACE2 in normal individuals or nodal metastasis status N0, N1, N2, N3 BRCA patients. (J) Relative expression of ACE2 in normal individuals at any age or BRCA patients aged 21–40, 41–60, 61–80, or 81–100 yrs. (K) Relative expression of ACE2 in normal individuals of any race or BRCA patients of Caucasian, African‐American, or Asian race. (*p < 0.05, **p < 0.01, ***p < 0.001)

**FIGURE 3:**
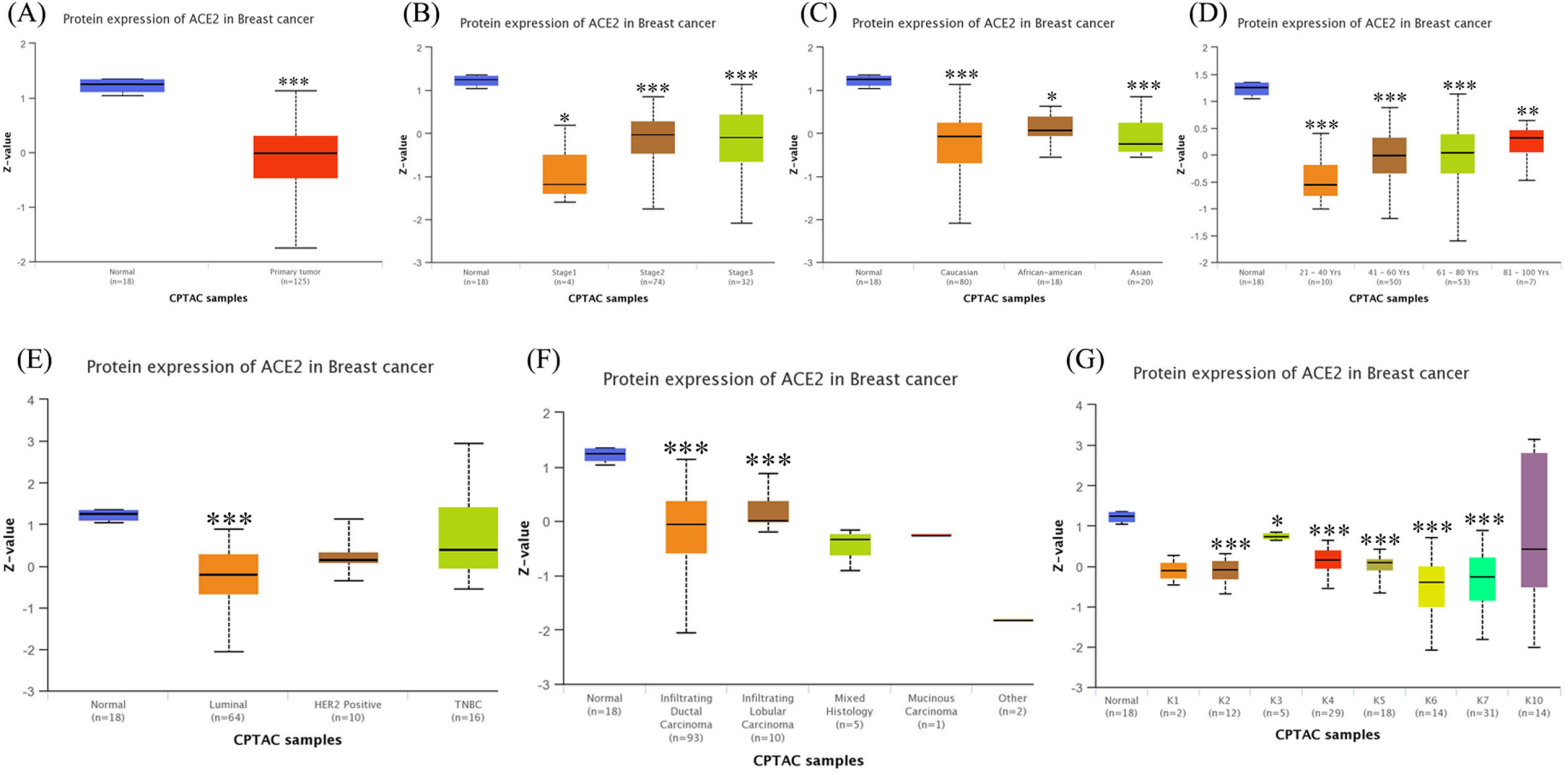
The protein expression levels of ACE2 in BRCA (UALCAN). (A) ACE2 protein expression in normal or breast cancer patients. (B) ACE2 protein expression in normal individuals or stage 1, 2, or 3 breast cancer patients. (C) ACE2 protein expression in normal individuals of any race or breast cancer patients of Caucasian, African‐American, or Asian race. (D) ACE2 protein expression in normal individuals at any age or breast cancer patients aged 21–40, 41–60, 61–80, or 81–100 years. (E) ACE2 protein expression in normal individuals or Luminal, HER2 positive, or Triple‐negative breast cancer (TNBC) patients. (F) ACE2 protein expression in normal individuals or patients with IDC, ILC, Mixed histology, Mucinous Carcinoma, Medullary Carcinoma, or other types of breast cancer. (G) ACE2 protein expression in normal individuals or breast cancer patients with pan‐cancer subtypes of cancer (K1 to K10). (*p < 0.05, **p < 0.01, ***p < 0.001)

We initially analyzed the Oncomine database to study differential ACE2 expression between various cancers and normal tissues. From the results, an abnormal expression level of ACE2 was observed in breast, colorectal, renal, and lung cancers, as well as sarcoma. For breast cancer, ACE2 expression increased particularly in Invasive Breast Carcinoma Stroma and decreased in IDC and ILC (Figure 1A). Then, we evaluated the transcription and protein expression level of ACE2 in BRCA using the Oncomine and UALCAN databases. Both databases showed that samples from BRCA patients had a significantly lower level of ACE2 compared to normal tissues (*p *< 0.001) (Figure 1B–E). Furthermore, analysis of numerous clinicopathological features of BRCA samples in the TCGA showed that *ACE2* mRNA expression mainly correlates with gender, histologic subtypes, cancer stages, TP53 mutation status, menopause status, and cancer subclasses (Figure 2). By CPTAC analysis, ACE2 protein expression decreased significantly on the basis of age, race, stages, major subclass, tumor histology, and Pan‐cancer subtype (Figure 3).

### 3.2 The methylation level of ACE2 promoter decreased in BRCA

**FIGURE 4:**
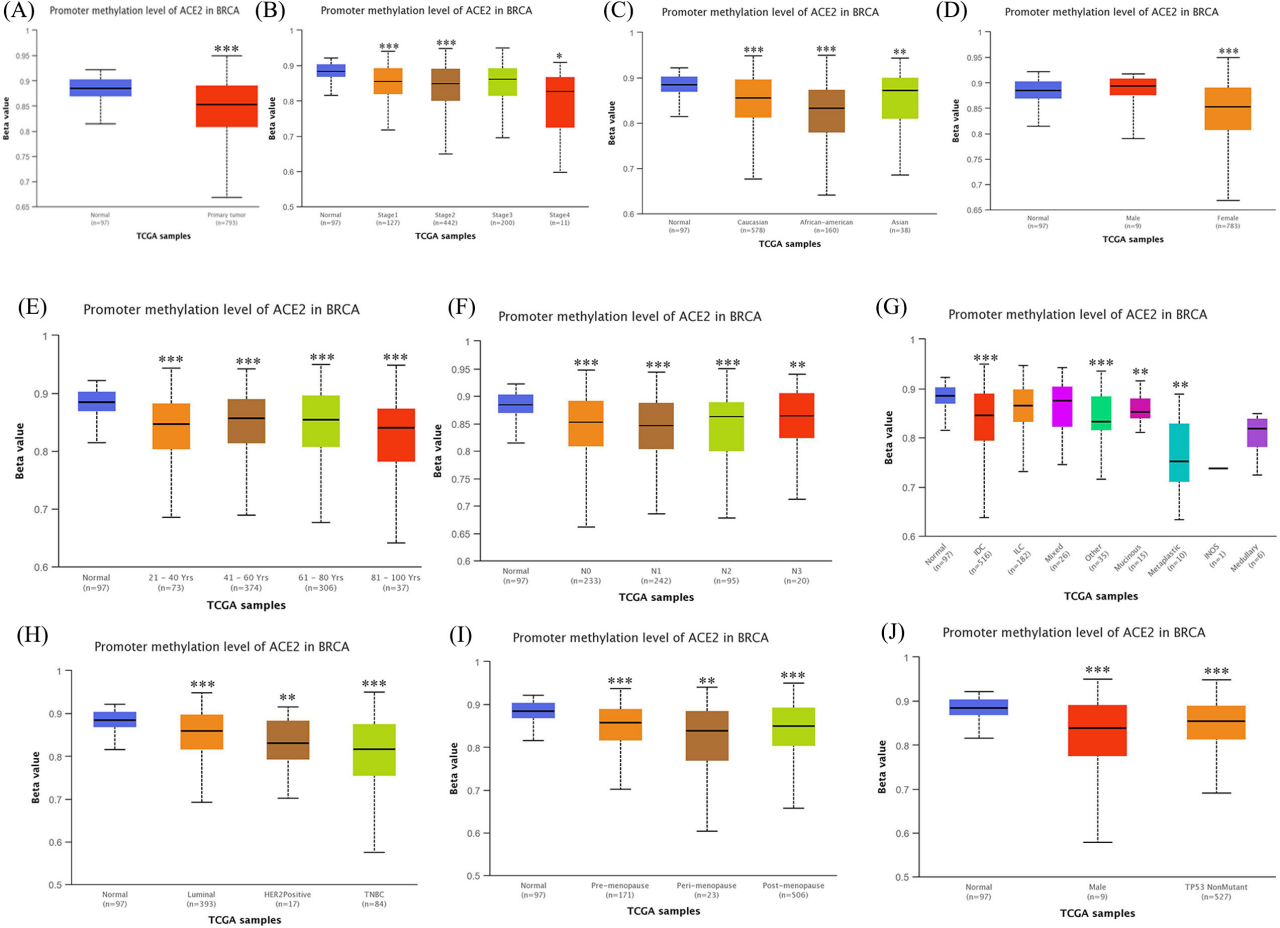
*ACE2* promoter methylation levels in BRCA (UALCAN). (A) *ACE2* promoter methylation levels in normal or BRCA samples. (B) *ACE2* promoter methylation levels in normal individuals or stage 1, 2, 3, or 4 breast cancer patients. (C) *ACE2* promoter methylation levels in normal individuals of any race or breast cancer patients of Caucasian, African‐American, or Asian race. (D) *ACE2* promoter methylation levels in normal individuals or male or female BRCA patients. (E) *ACE2* promoter methylation levels in normal individuals at any age or breast cancer patients aged 21–40, 41–60, 61–80, or 81–100 yrs. (F) *ACE2* promoter methylation levels in normal individuals or nodal metastasis status N0, N1, N2, N3 BRCA patients. (G) *ACE2* promoter methylation levels in normal individuals or patients with IDC, ILC, Mixed histology, Mucinous Carcinoma, Mucinous Carcinoma, Infiltrating Carcinoma NOS (INOS), Metaplastic Carcinoma, Medullary Carcinoma, or any other type of BRCA. (H) *ACE2* promoter methylation levels in normal individuals or Luminal, HER2 positive, or TNBC patients. (I) *ACE2* promoter methylation levels in normal individuals or Pre‐Menopause, Peri‐Menopause, or Post‐Menopause BRCA patients. (J) *ACE2* promoter methylation levels in normal individuals or TP53‐Mutant or TP53‐NonMutant BRCA patients. (*p < 0.05, **p < 0.01, ***p < 0.001)

DNA promoter methylation usually inhibits gene transcription. To explore the influence of *ACE2* methylation on ACE2 expression in BRCA, we first determined the *ACE2* promoter methylation level in BRCA by utilizing the TCGA analysis module of the UALCAN database. ACE2 promoter methylation significantly reduced ACE2 expression, and this decrease was dependent on different clinicopathological features (Figure 4).

### 3.3 ACE2 served as a potential prognostic biomarker in BRCA

**FIGURE 5:**
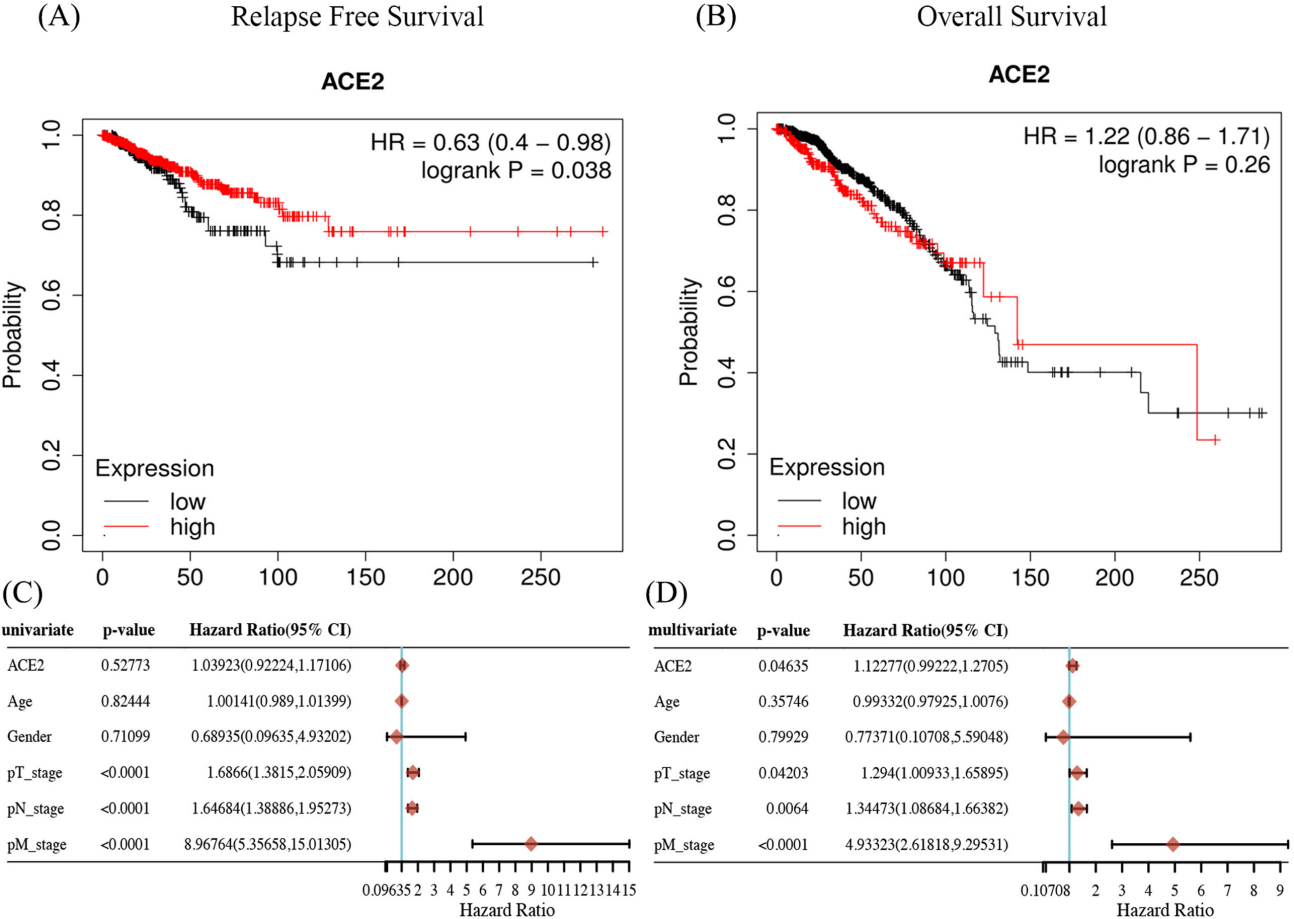
Kaplan‐Meier survival curves obtained by comparing the high and low expression of ACE2 in BRCA in the Kaplan‐Meier plotter database. (A) The relapse‐free survival (RFS) curve with respect to ACE2 expression in BRCA. (B) The overall survival (OS) curve with respect to ACE2 expression in BRCA. (C and D) Univariate and multivariate analysis of ACE2 expression and clinical parameters in BRCA

By using KMPD, we investigated the effect of ACE2 expression on RFS and OS of BRCA patients. BRCA patients with elevated ACE2 expression had a better prognosis, but the OS rate was not affected (Figure 5A,B). Further univariate and multivariate analysis revealed that ACE2 expression, pT stage, pN stage, and pM stage were independent factors affecting the prognosis of BRCA patients (Figure 5C,D). To better unveil the potential mechanism of ACE2 in BRCA, we evaluated the association of ACE2 expression and multiple clinicopathological factors in the Kaplan–Meier plotter databases. ACE2 overexpression was related to better RFS in stage 2 (HR = 0.49 (0.25–0.98), *p *= 0.04) and 3 (HR = 2.22 (1.13–4.37), *p *= 0.018) BRCA patients. In addition, high mutation burden might be a potential factor affecting BRCA prognosis (HR = 0.53 (0.26–1.08), *p *= 0.074) (Table 1).

**TABLE 1:** Correlation of ACE2 expression and the relapse‐free survival of BRCA with different clinicopathological factors (Kaplan‐Meier plotter)

Pathological parameters	Case number	Hazard radio	*p*‐Value
Stage status			
1	253	3.16 (0.39–25.38)	0.25
2	749	0.49 (0.25–0.98)	0.04
3	265	2.22 (1.13–4.37)	0.018
4	NA	NA	NA
Gender			
Female	1277	0.61 (0.4–0.96)	0.029
Male	NA	NA	NA
Race			
White	928	9.29 (0.78–110.02)	0.035
Asian	87	1109935981.52 (0‐Inf)	0.27
Black/African‐American	213	0.52 (0.21–1.29)	0.15
Mutation burden			
High	574	0.53 (0.26–1.08)	0.074
Low	584	0.65 (0.34–1.23)	0.18

### 3.4 ACE2 was related to immune infiltration in BRCA

**FIGURE 6:**
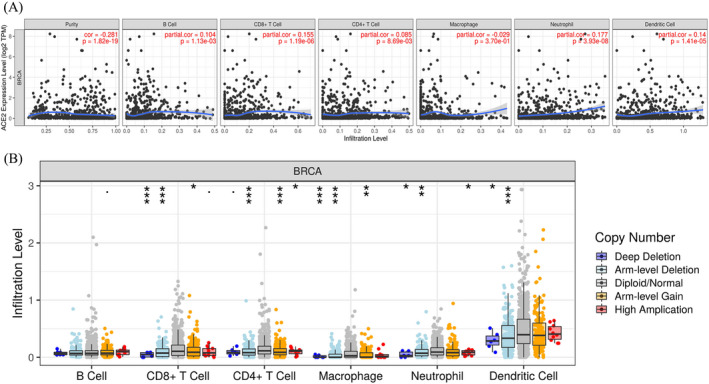
Correlation of ACE2 expression with immune infiltration level in BRCA (TIMER). (A) ACE2 expression is significantly negatively correlated to tumor purity and has significant positive correlations with infiltrating levels of B cells, CD8^+^ T cells, CD4^+^ T cells, neutrophils, and dendritic cells in BRCA, but not with that of macrophages. (B) Copy number mutation of ACE2 in BRCA could partly inhibit the immune infiltration level of CD8^+^ T cells, CD4^+^ T cells, neutrophils, macrophages, and dendritic cells, but not of B cells (*p*‐value Significant Codes: 0 ≤ *** <0.01 ≤ * <0.05≤. <0.1)

Substantial studies have reported that ACE2 plays key roles in immunity.^15^ Therefore, we adopted the TIMER database for exploring the link between ACE2 expression and immune infiltration level in BRCA. From the results, we observed that ACE2 expression levels increased as the abundance of immune cells, including B cells, CD8^+^ T cells, CD4^+^ T cells, Neutrophils, and Dendritic cells, increased (Figure 6A, all *p *< 0.05). In addition, copy number mutation of ACE2 could partly inhibit the immune infiltration level of these immune cells except for B cells (Figure 6B). We then explored the link between ACE2 expression and the gene biomarkers of distinct immune cells in BRCA. The immune biomarkers of CD8^+^ T cells (CD8A and CD8B), T cells (CD3D, CD3E, and CD2), B cells (CD19 and CD79A), Monocytes (CD86), TAM (CCL2 and IL‐10), M1 Macrophages (INOS and COX2), M2 Macrophages (CD163 and MS4A4A), Neutrophils (CD66b and CCR7) NK cells (KIR2DL1, KIR2DL3, KIR2DL4, KIR3DL1, KIR3DL2, KIR3DL3, and KIR2DS4), Dendritic cell (HLA‐DQB1, HLA‐DRA, and BDCA‐4), Th1 (T‐bet, STAT4, STAT1, IFN‐g, and TNF‐a), Th2 (GATA3, STAT6, STAT5A, and IL13), Tfh (IL21), Th17 (IL17A), Treg (FOXP3, CCR8, STAT5B, and TGFb), T‐cell exhaustion (PD‐1, CTLA4, LAG3, and GZMB) were significantly associated with ACE2 expression level in BRCA after purity adjustment (Table 2). Thus, all these results suggest a strong relationship between ACE2 and immune infiltrating cells. Moreover, ACE2 expression may hold the key to the immune response in BRCA, indicating the potential of ACE2 as a target for BRCA immunotherapy.

**TABLE 2:** Correlation analysis between ACE2 and gene biomarkers of immune cells in BRCA (TIMER)

Description	Biomarkers	None		Purity	
		Cor	*p*‐Value	Cor	*p*‐Value
CD8^+^ T cell	CD8A	0.0226	3.36e‐14	0.116	2.42e‐04
	CD8B	0.284	8.06e‐22	0.191	1.21e‐09
T cell (general)	CD3D	0.265	3.54e‐19	0.146	3.67e‐06
	CD3E	0.264	4.62e‐19	0.145	4.36e‐06
	CD2	0.264	5.76e‐19	0.154	1.07e‐06
B cell	CD19	0.273	3.26e‐20	0.156	7.14e‐07
	CD79A	0.283	9.33e‐22	0.163	2.43e‐07
Monocyte	CD86	0.148	7.71e‐07	0.06	5.69e‐02
	CD115(CSF1R)	0.102	6.63e‐04	‐0.016	6.18e‐01
TAM	CCL2	0.293	3.09e‐23	0.228	3.48e‐13
	CD68	0.093	2.04e‐03	0.006	8.42e‐01
	IL10	0.166	2.91e‐08	0.096	2.36e‐03
M1 Macrophage	INOS (NOS2)	0.09	2.71e‐03	0.063	4.54e‐02
	IRF5	0.009	7.7e‐01	‐0.048	1.33e‐01
	COX2(PTGS2)	0.402	6.47e‐44	0.331	6.61e‐27
M2 Macrophage	CD163	0.173	7.7e‐09	0.11	5.06e‐04
	VSIG4	0.107	3.61e‐04	0.032	3.14e‐01
	MS4A4A	0.173	8.18e‐09	0.086	6.42e‐03
Neutrophils	CD66b (CEACAM8)	0.069	2.17e‐02	0.074	2.02e‐02
	CD11b (ITGAM)	0.066	2.94e‐02	‐0.021	5.03e‐01
	CCR7	0.244	2.1e‐16	0.128	5.26e‐05
Natural killer cell	KIR2DL1	0.165	3.47e‐08	0.112	4.15e‐04
	KIR2DL3	0.188	3.39e‐10	0.115	2.74e‐04
	KIR2DL4	0.273	2.83e‐20	0.207	4.17e‐11
	KIR3DL1	0.188	3.42e‐10	0.114	3.34e‐04
	KIR3DL2	0.234	3.92e‐15	0.15	2.08e‐06
	KIR3DL3	0.125	3.08e‐05	0.092	3.75e‐03
	KIR2DS4	0.219	2.34e‐13	0.151	1.79e‐06
Dendritic cell	HLA‐DPB1	0.138	4.11e‐06	‐0.017	5.89e‐01
	HLA‐DQB1	0.169	1.82e‐08	0.071	2.47e‐02
	HLA‐DRA	0.19	1.99e‐10	0.068	3.18e‐02
	HLA‐DPA1	0.128	2.12e‐05	‐0.007	8.24e‐01
	BDCA‐1(CD1C)	0.179	2.28e‐09	0.032	3.13e‐01
	BDCA‐4(NRP1)	0.151	4.62e‐07	0.078	1.37e‐02
	CD11c (ITGAX)	0.118	9.11e‐05	0.003	9.35e‐01
Th1	T‐bet (TBX21)	0.285	5.55e‐22	0.185	4.10e‐09
	STAT4	0.303	8.14e‐25	0.201	1.5e‐10
	STAT1	0.154	296e‐07	0.127	5.62e‐05
	IFN‐g (IFNG)	0.275	1.33e‐20	0.196	4.15e‐10
	TNF‐a (TNF)	0.172	9.72e‐09	0.123	9.83e‐05
Th2	GATA3	‐0.553	4.88e‐89	‐0.522	1.69e‐70
	STAT6	‐0.112	2e‐04	‐0.151	1.80e‐06
	STAT5A	0.149	6.68e‐07	0.082	9.81e‐03
	IL13	0.212	1.11e‐12	0.16	3.74e‐07
Tfh	BCL6	0.076	1.2e‐02	0.027	3.96e‐01
	IL21	0.202	1.28e‐11	0.15	2.06e‐06
Th17	STAT3	0.036	2.37e‐01	0.017	6.01e‐01
	IL17A	0.194	8e‐11	0.158	5.38e‐07
Treg	FOXP3	0.282	1.75e‐21	0.187	2.64e‐09
	CCR8	0.173	7.24e‐09	0.109	5.97e‐04
	STAT5B	‐0.061	4.27e‐02	‐0.093	3.49e‐03
	TGFb (TGFB1)	‐0.063	3.77e‐02	‐0.196	4.88e‐10
T cell exhaustion	PD‐1 (PDCD1)	0.272	4.25e‐20	0.172	5.30e‐08
	CTLA4	0.326	1.4e‐28	0.244	5.67e‐15
	LAG3	0.252	2.39e‐17	0.201	1.65e‐10
	TIM‐3 (HAVCR2)	0.087	3.98e‐03	0.002	9.39e‐01
	GZMB	0.356	4.06e‐34	0.281	1.83e‐19

### 3.5 Prognostic value of ACE2 expression in BRCA

According to the results of section 3.4, ACE2 affects both the prognosis and the immune infiltration in BRCA. Moreover, immune infiltration affects BRCA prognosis. However, whether ACE2 affects the BRCA prognosis through immune infiltration is unknown. Hence, we adopted the KMPD for analyzing the BRCA prognosis on basis of the expression level of ACE2 in related immune cell subsets. The results revealed that ACE2 levels were enriched in basophils (HR = 0.56, *p *= 0.021) and CD8+ T‐cells (HR = 0.56, *p *= 0.03) and type‐2 T‐helper cells (HR = 0.48, *p*=0.02), which showed decreasing numbers and positively correlated with BRCA prognosis (Figure S1). However, there was no significant association between these factors in B cell, CD4^+^ memory T‐cell, eosinophils, macrophage, Mesenchymal stem cell, NK T‐cell, and regulatory and Type 1 helper T‐cells (Figure S2). Thus, the results indicate that ACE2 affects the prognosis of BRCA partly through immune infiltration.

### 3.6 Enrichment analysis of ACE2 in BRCA

**FIGURE 7:**
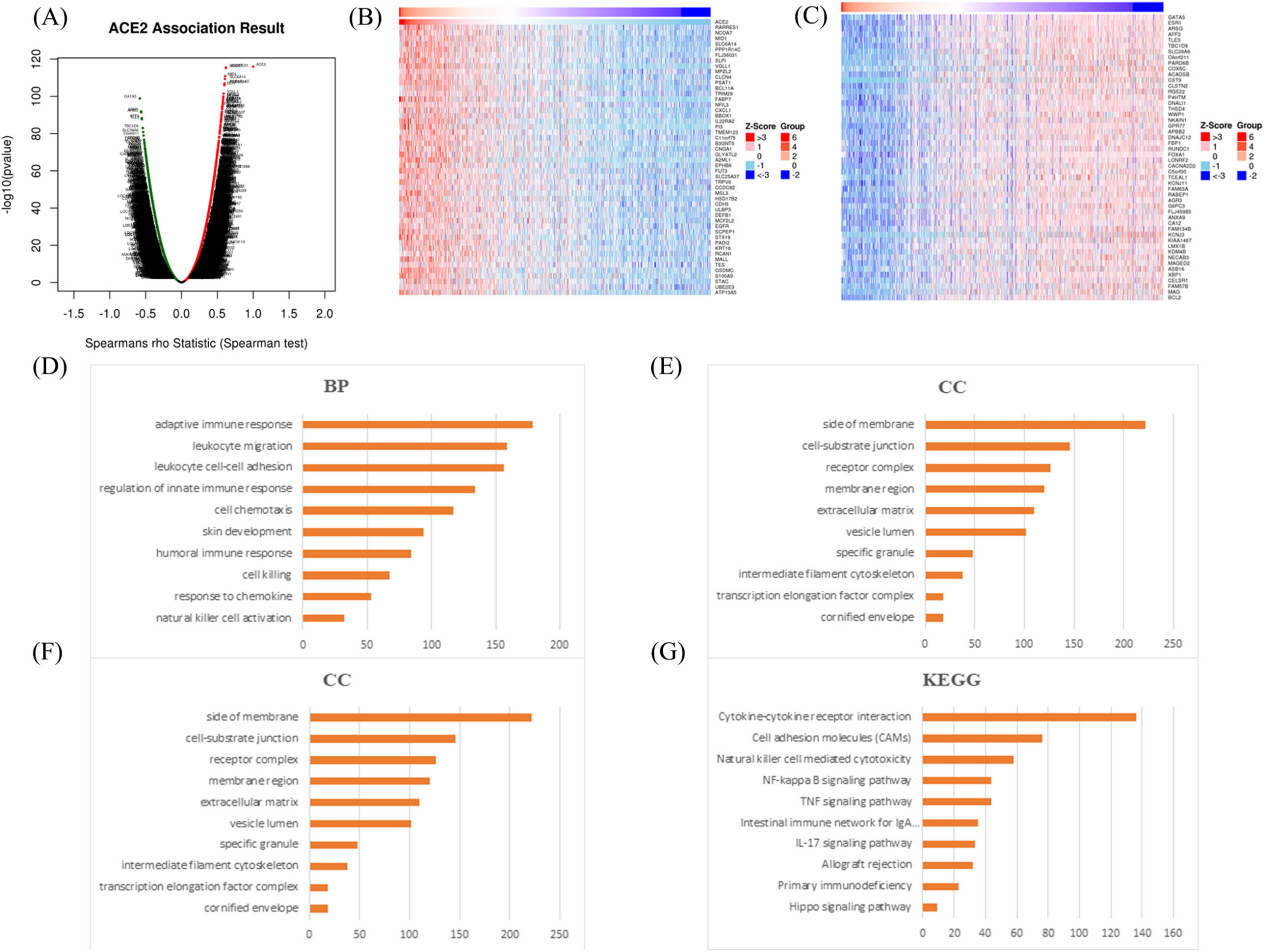
Genes related to ACE2 and enrichment analyses in BRCA (LinkedOmics). (A) Volcano plot showing ACE2 and associated genes’ expression in BRCA by using Spearman test (*n =* 1093). Dark red dots represent genes positively correlated with ACE2 and dark green dots represent genes negatively correlated with ACE2. (B) Heat map showing top 50 genes positively correlated with ACE2 in BRCA. (C) Heat map showing top 50 genes negatively correlated with ACE2 in BRCA

According to the mRNA sequencing data of 1093 TCGA BRCA patients from LinkedOmics, we discovered that 8628 genes were positively correlated while 5817 genes were negatively correlated with ACE2 expression (Figure 7A–C). show the top 50 genes positively and negatively correlated with ACE2, respectively. GO enrichment analysis of ACE2 using GSEA revealed that terms related to adaptive immune response, regulation of innate immune response, cell chemotaxis, cell‐substrate junction, and receptor‐ligand activity were enriched (Figure 7D–F). Moreover, according to KEGG enrichment analysis, ACE2 was demonstrated to be involved in NF‐κB, IL‐17, and TNF signaling pathways (Figure 7G).

### 3.7 ACE2‐associated kinase, miRNA, or transcription factor targets in BRCA

Given the significance of ACE2 in BRCA, the kinase, miRNA, and transcription factor networks of ACE2 in BRCA were further analyzed with LinkedOmics by utilizing GSEA (Table 3). The top five most important kinase target networks were found to be Mitogen‐activated protein kinase 8 (MAPK8), Protein Kinase C Alpha (PRKCA), PAK3 (P21 (RAC1) Activated Kinase 3), PAK5 (P21 (RAC1) Activated Kinase 5), and Pyruvate Kinase M1/2 (PKM). The miRNA target network involved the following miRNAs: (ATGTACA) MIR‐493, (GTCAACC) MIR‐380‐5P, (ACAGGGT) MIR‐10A, and MIR‐10B, (TACTTGA) MIR‐26A and MIR‐26B, and (AATGTGA) MIR‐23A and MIR‐23B. The target network of transcription factors included V$IRF_Q6, V$NFKB_Q6_01, V$SRF_Q4, V$PBX1_02, and V$CEBPB_02.

**TABLE 3:** The kinase, miRNA and transcription factor‐target networks of ACE2 in BRCA (LinkedOmics)

Enriched category	Geneset	Leading Edge Numer	*p* Value
Kinase target	Kinase_MAPK8	14	0.0022075
	Kinase_PRKCA	98	0.0031746
	Kinase_PAK3	7	0.0031746
	Kinase_PAK5	7	0.0046729
	Kinase_PKM	2	0.0054054
miRNA target	ATGTACA, MIR‐493	63	0
	GTCAACC, MIR‐380‐5P	8	0.0030769
	ACAGGGT, MIR‐10A, MIR‐10B	38	0.0047619
	TACTTGA, MIR‐26A, MIR‐26B	73	0.012739
	AATGTGA, MIR‐23A, MIR‐23B	86	0.014553
Transcription factor target	V$IRF_Q6	74	0
	V$NFKB_Q6_01	56	0
	V$SRF_Q4	78	0
	V$PBX1_02	28	0
	V$CEBPB_02	54	0

### 3.8 Validation of the expression and prognostic value of ACE2 in breast cancer

**FIGURE 8:**
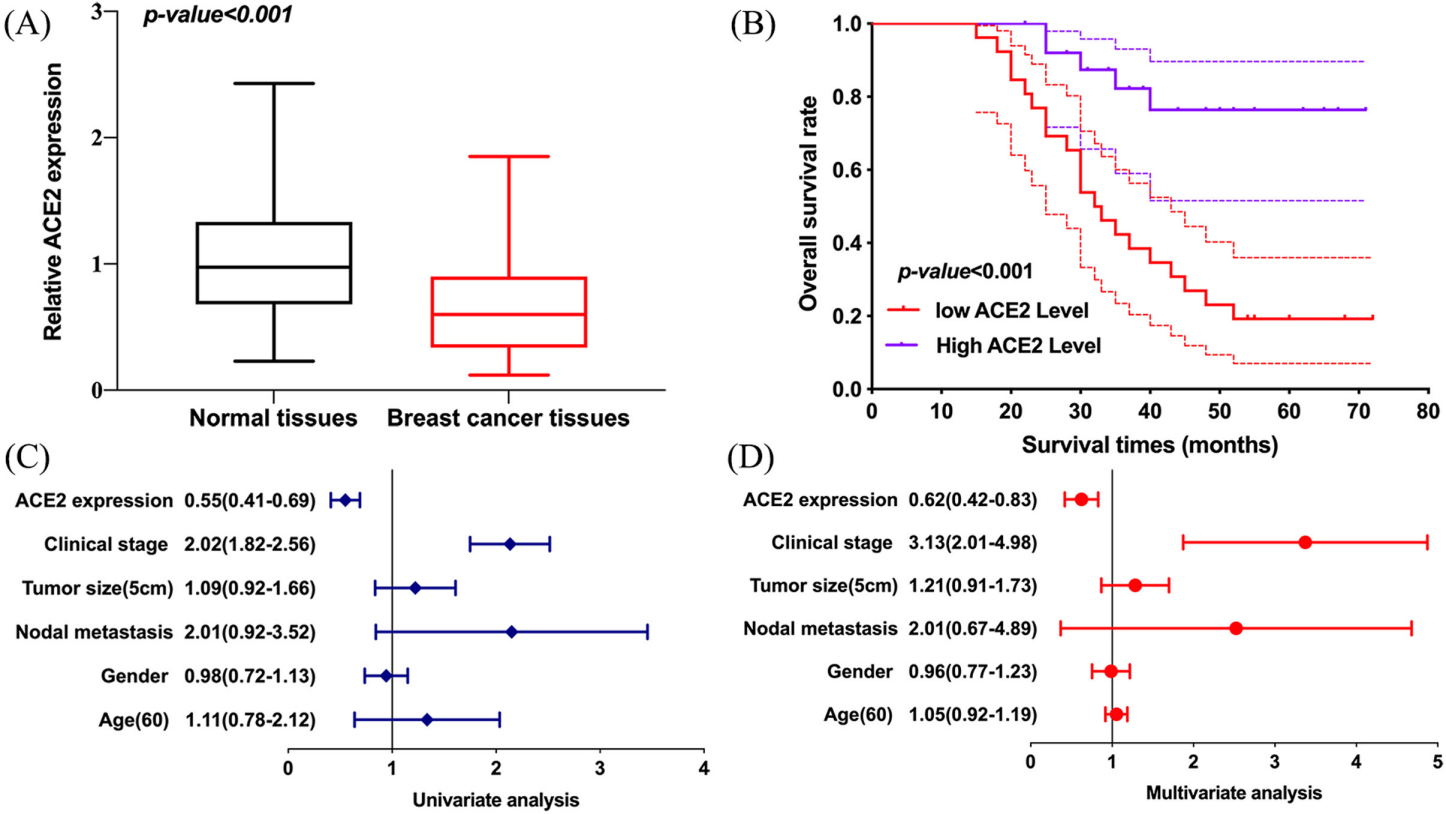
The expression and prognosis value of ACE2 in breast cancer. (A) The relative expression of ACE2 in breast cancer tissues versus normal tissues. (B) Overall survival curve of high and low ACE2 expression group. (C and D) Univariate and multivariate analysis considering clinical parameters and ACE2 expression in breast cancer

qRT‐PCR was performed to verify the expression and prognostic value of ACE2 in breast cancer using the clinical tissues from Taizhou First People's Hospital (*N =* 52). As shown in Figure 8A, ACE2 was downregulated in breast cancer versus normal tissues (*p *< 0.001). Moreover, OS analysis indicated a better prognosis in breast cancer patients with high ACE2 expression (*p *< 0.001, Figure 8B). As shown in Figure 8C,D, the univariate and multivariate analysis also demonstrated ACE2 expression and clinical stage as independent prognostic factors for breast cancer. These data further confirm the prognostic value of ACE2 in BRCA.

## 4 DISCUSSION

Recent studies have reported that ACE2 levels negatively correlate with tumor grade and the ability to metastasize.^16^ In UCEC (Uterine Corpus Endometrial Carcinoma) and KIRP (Kidney Renal Papillary Cell Carcinoma), ACE2 expression shows a positive correlation with the prognosis, accompanied by elevated immune cell infiltration levels.^17^ As for SARS‐CoV‐2 infection, ACE2 together with TMPRSS2 acts as a receptor for host cell entry.^18^ In this study, Oncomine and UALCAN databases were used to assess ACE2 expression levels. RFS and OS survival analyses were performed in the KMPD. We also explored the interaction between ACE2 expression and immune cell infiltration via the TIMER database. Moreover, the LinkedOmics database was utilized for enrichment analyses.

From the results, we observed that ACE2 expression levels decreased in breast cancer, especially BRCA. ACE2 may serve as a probable prognosis factor for BRCA, partly by affecting immune infiltration. ACE2 performs both positive and negative roles in cancer therapies. Low ACE2 expression level is commonly a sign of cancer presence along with diabetes.^19^ In several cancers such as pancreatic, lung, colon, and breast cancers, ACE2 inhibits metastasis and angiogenesis.^20^ Specifically, in hepatocellular carcinoma (HCC) patients, increased ACE2 expression reflects a longer survival time compared to those with decreased ACE2 expression, which implies the prognosis role of ACE2 in HCC.^21^ Likewise, ACE2 has been pointed out as a prognostic factor in gallbladder carcinoma.^22^ Several other factors including age, gender, and health status of cancer patients need to be considered as well.^23^

The human epidermal growth factor receptor (HER2), estrogen receptor (ER), and progesterone receptor (PR) are the most meaningful classical biomarkers in all breast cancer patients.^24^ Moreover, *BRCA1* and *BRCA2* are genomic markers, encoding large proteins acting as tumor suppressor products that affect transcription and regulate the cell cycle, thus protecting DNA during replication. BRCA1/2 mutations are responsible for almost half of hereditary breast cancers.^25^ Immune infiltration largely influences the prognosis and clinical outcome of breast cancer. T regulatory cells (Tregs), M0 macrophages, and M2 macrophages, contributing to tumorigenesis, are associated with a poor prognosis regardless of ER status. M1 macrophages are anti‐tumoral and M2 macrophages are pro‐tumoral. Lack of immune infiltration is correlated to the worst prognosis of ER‐negative breast cancer and intermediate prognosis of ER‐positive disease.^26^

Our KEGG enrichment analysis of ACE2 in BRCA showed that IL‐17 and NF‐κB signaling pathways are of major importance. IL‐17 family proteins (IL17A to IL17F), secreted by T helper 17 (Th17) cells, are effective in several inflammatory diseases and tumors.^27^ IL‐17 cytokines family can activate the production of NF‐κB, IL8, IL6, TNF‐α, and IL1b, contributing to various inflammatory diseases and cancers.^28^ Recently, another study shows that high expression of IL17B and its receptor IL17RB relates to poor prognosis of breast cancer patients.^29^ Moreover, Protein kinase C α (PKCα) has been reported to contribute to the metastasis and aggravation of breast cancer. Specifically, high expression levels of PKCα and FOX2 are found in TNBC tumors.^30^ Activation of PKCα is reported to enhance the internalization of HER2.^31^ In breast cancer patients, miR‐23A is involved in the regulation of autophagy pathways, implying a future potential therapeutic target and biomarker.^32^

To explore the reasons for reduced ACE2 expression in BRCA, we investigated the DNA methylation level of *ACE2* in BRCA. Usually, high expression levels of ACE2 in tumors, including colon adenocarcinoma (COAD), Kidney renal papillary cell carcinoma (KIRP), pancreatic cancer (PAAD), rectal adenocarcinoma (READ), and lung adenocarcinoma (LUAD), are always accompanied by low DNA methylation level of *ACE2*.^33^ Surprisingly, in our study, the ACE2 expression level was found to decrease after DNA promoter methylation. *ACE2* may be inactivated and downregulated due to hypermethylation, which may partly explain the decreased ACE2 expression in BRCA.

Our study had a few limitations. First, most analyses were performed at the mRNA level but not at the protein and gene level. Furthermore, our results need to be validated by performing in vivo and in vitro experiments. In addition, histochemistry staining should be performed to verify the expression of ACE2 in BRCA.

## 5 CONCLUSION

In conclusion, ACE2 expression declined in BRCA, which to some extent resulted from *ACE2* methylation. ACE2 exhibited good prognostic value and was associated with better RFS and immune infiltration in BRCA. Thus, ACE2 may serve as a potential biomarker and therapeutic target for BRCA.

## CONFLICT OF INTEREST

The authors declare no conflicts of interests.

## Supporting information

Figure S1

Figure S2

## Data Availability

The analyzed datasets generated during the study are available from the corresponding author on reasonable request.
