# Supplementary figures and images for: ACE2 maybe serve as a prognostic biomarker in breast invasive carcinoma
Source: J Clin Lab Anal. 2022 Apr 4;36(6):e24362. doi: 10.1002/jcla.24362 (PMC9169220; doi:10.1002/jcla.24362)

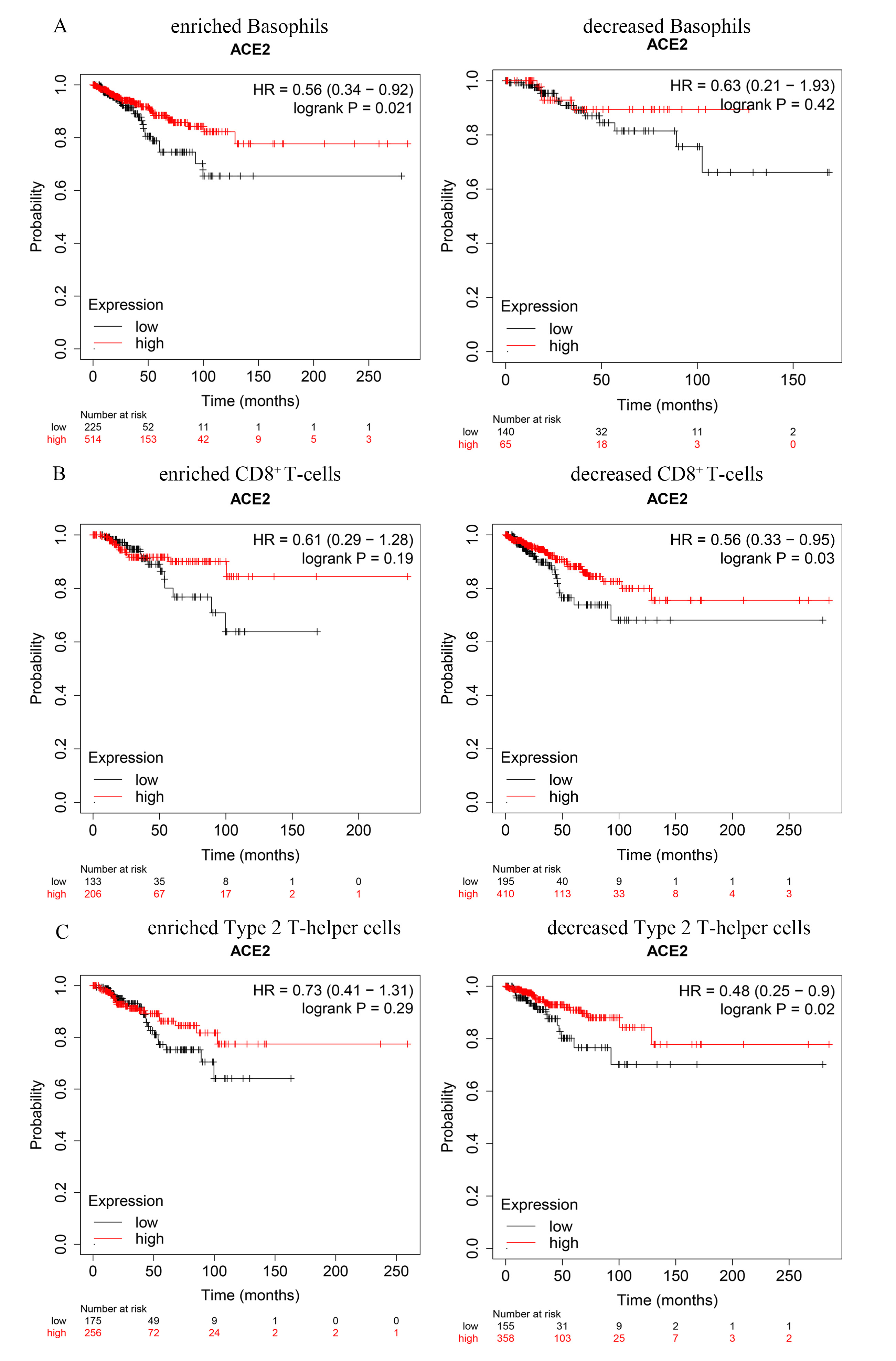

Supplement: Supplementary file 1 — Figure S1 [file JCLA-36-e24362-s001.jpg]

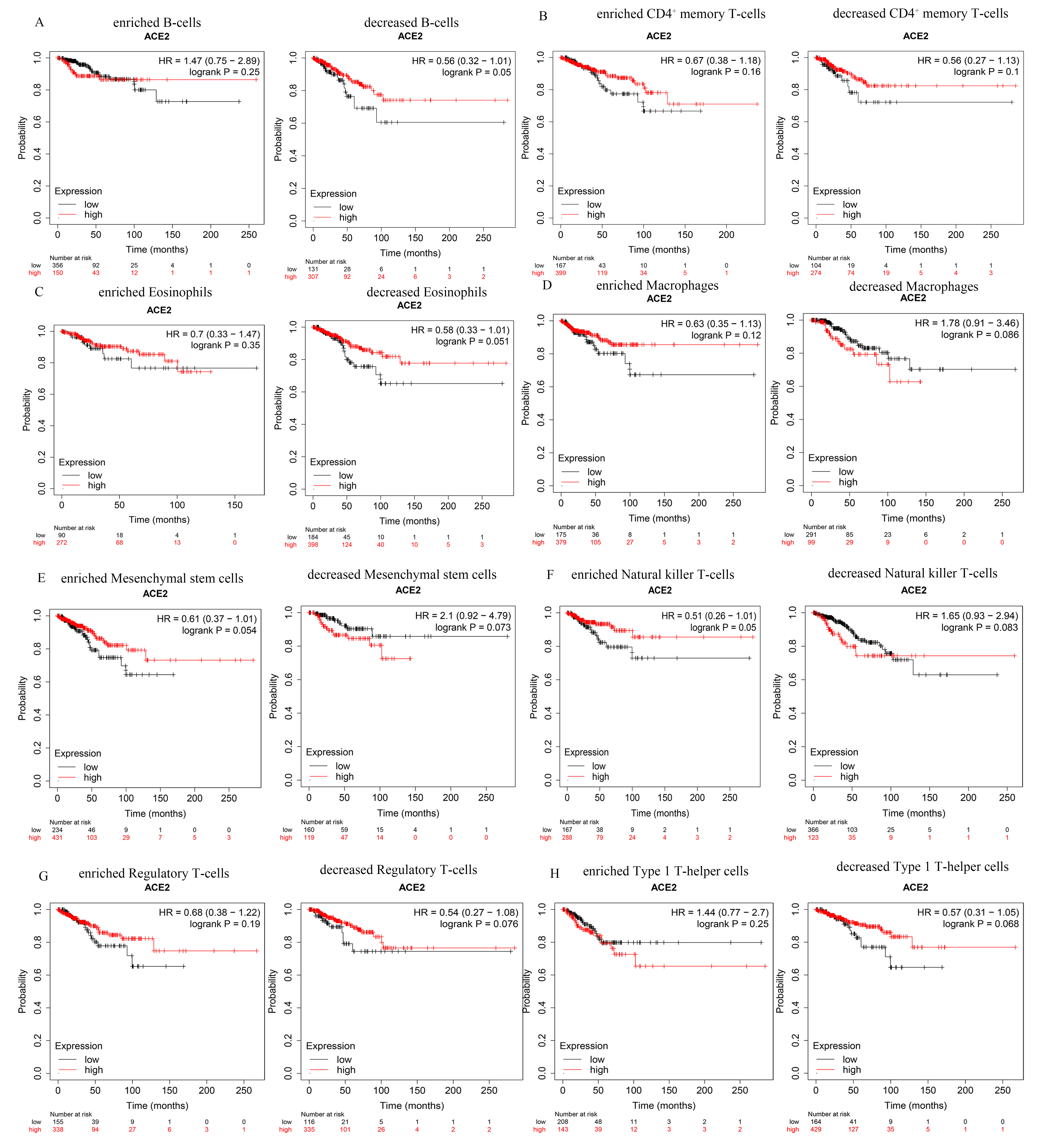

Supplement: Supplementary file 2 — Figure S2 [file JCLA-36-e24362-s002.jpg]
